# New, Fully Implantable Device for Selective Clearance of CSF-Target Molecules: Proof of Concept in a Murine Model of Alzheimer’s Disease

**DOI:** 10.3390/ijms23169256

**Published:** 2022-08-17

**Authors:** María Almudena Coto-Vilcapoma, Juan Castilla-Silgado, Benjamín Fernández-García, Paola Pinto-Hernández, Raffaela Cipriani, Estibaliz Capetillo-Zarate, Manuel Menéndez-González, Marco Álvarez-Vega, Cristina Tomás-Zapico

**Affiliations:** 1 Departamento de Biología Funcional, Área de Fisiología, Universidad de Oviedo, 33006 Oviedo, Spain; 2 Instituto de Investigación Sanitaria del Principado de Asturias, 33011 Oviedo, Spain; 3 Departamento de Morfología y Biología Celular, Área de Anatomía, Universidad de Oviedo, 33006 Oviedo, Spain; 4 Achucarro Basque Center for Neuroscience, Departamento de Neurociencias, Universidad del País Vasco (UPV/EHU), 48940 Leioa, Spain; 5 Centro de Investigación en Red de Enfermedades, Neurodegenerativas (CIBERNED), 28029 Madrid, Spain; 6 IKERBASQUE, Basque Foundation for Science, 48009 Bilbao, Spain; 7 Servicio de Neurología, Hospital Universitario Central de Asturias, 33011 Oviedo, Spain; 8 Departamento de Medicina Área de Medicina, Universidad de Oviedo, 33006 Oviedo, Spain; 9 Servicio de Neurocirugía, Hospital Universitario Central de Asturias, 33011 Oviedo, Spain; 10 Departamento de Cirugía, Área de Cirugía, Universidad de Oviedo, 33006 Oviedo, Spain

**Keywords:** immunotherapy, Alzheimer, beta-amyloid, CSF-sink, cerebrospinal fluid, implantable device, blood–brain barrier, nanoporous membranes

## Abstract

We have previously proposed a radical change in the current strategy to clear pathogenic proteins from the central nervous system (CNS) based on the *cerebrospinal fluid (CSF)-sink* therapeutic strategy, whereby pathogenic proteins can be removed directly from the CNS via CSF. To this aim, we designed and manufactured an implantable device for selective and continuous apheresis of CSF enabling, in combination with anti-amyloid-beta (Aβ) monoclonal antibodies (mAb), the clearance of Aβ from the CSF. Here, we provide the first proof of concept in the APP/PS1 mouse model of Alzheimer’s disease (AD). Devices were implanted in twenty-four mice (seventeen APP/PS1 and seven Wt) with low rates of complications. We confirmed that the apheresis module is permeable to the Aβ peptide and impermeable to mAb. Moreover, our results showed that continuous clearance of soluble Aβ from the CSF for a few weeks decreases cortical Aβ plaques. Thus, we conclude that this intervention is feasible and may provide important advantages in terms of safety and efficacy.

## 1. Introduction

The social impact of neurodegenerative diseases (NDD) is undeniable. They comprise a wide range of different neuropathologies, which can be either sporadic or inherited. However, most of them share the common hallmark of deposits of disease-specific proteins; thus, NDD can be understood as proteinopathies.

Interestingly, in the symptomatic Huntington’s disease (HD) mouse model, blocking mutant huntingtin expression favors the disappearance of aggregates and ameliorates the behavioral phenotype [1]. This opened an insight into the pathophysiology of NDD where protein aggregation overwhelms the proteostasis capacity of neurons (e.g., ubiquitin-proteasome and autophagy-lysosome systems), interfering with the ability of neurons to cope with pathogenic proteins [2,3]. The formation of aggregates of these proteins can have different origins, including, among others, increased synthesis or synthesis of structurally abnormal forms and/or decreased degradation, either by enzymatic or cellular systems [4,5]. A decrease in their clearance to compartments outside the brain parenchyma has also been described, which has been linked to the impairment of the blood–brain barrier (BBB) [6], the cerebrospinal fluid (CSF) flow [7], and the glymphatic system [8,9]. 

Accumulation of amyloid-beta (Aβ) is the main pathological hallmark of Alzheimer’s disease (AD). In both early-onset and late-onset forms of AD, Aβ clearance seems already impaired at the prodromal stage of AD, and it is removed from the brain by various overlapping and interacting clearance systems: degradation, BBB transport, interstitial fluid (ISF) bulk flow, the glymphatic pathway, and CSF absorption into the circulatory and lymphatic systems [10,11]. 

Different approaches have been investigated to remove Aβ both biologically and mechanically, from decreasing production (i.e., BACE inhibitors) to increasing clearance in the periphery (immunotherapy, plasmapheresis, enzymatic degradation) [12]. Among them, immunotherapy with anti-Aβ monoclonal antibodies (mAb) is the most extensively explored in humans, showing the capacity to clear brain plaques and restore levels of soluble Aβ in the CNS [13]. However, none of these therapies have shown clinically relevant benefits to AD patients, and serious side effects have been reported, including amyloid-related imaging abnormalities (ARIA) after anti-Aβ mAb therapies [14]. These failures led to seek alternative methods to eliminate pathogenic proteins from the brain using other chemical or physical principles, such as hemodialysis or plasmapheresis. Among the results obtained from the research on these interventions, it has been found that blood dialysis and plasmapheresis reduce Aβ levels in plasma and CSF in AD patients and attenuate AD symptoms and pathology in AD mouse models [15,16,17,18,19,20,21]. This suggests that removing Aβ from the plasma might be an effective form for generating an efflux of brain Aβ through the BBB [22].

The BBB prevents the free movement of molecules between the interstitial fluid (ISF)/CSF and plasma [11], while ISF soluble molecules move in constant equilibrium between the CSF and the ISF, both being compartments in direct communication [7,22]. Given this, we have previously proposed a radical change in the current paradigm based on clearing target molecules from the CSF using implantable devices [23]. Our hypothesis relies on the *CSF-sink* therapeutic strategy, whereby pathogenic proteins that are in equilibrium between ISF and CSF can be removed directly from CSF [24,25,26]. This involves an alteration of this equilibrium, favoring the clearance of these proteins in their soluble state and thus decreasing their availability to form aggregates in the brain parenchyma. Interestingly, the equilibrium between ISF and CSF remains stable in symptomatic AD models, in contrast to the balance loss between the ISF and plasma [22]. Thus, there is a much more direct way of removing target proteins from the ISF than clearing them from the plasma: clearing them from the CSF.

To this aim, we propose to use a procedure we named CSF apheresis based on an implantable device with a nanoporous system allowing selective and continuous apheresis of Aβ from the CSF. To evaluate the biological efficacy of the system, we have developed a miniaturized prototype to conduct studies in murine models of NDD. Here, we present the first proof of concept in the APP/PS1 mouse model of AD.

## 2. Results

### 2.1. Implantation of the Miniaturized Prototype Was Feasible in a Murine Model of Alzheimer’s Disease

For this proof of concept, we have used a well-characterized murine model of AD for which the time at which Aβ plaques can be observed in the brain is well known (six months) [27]. To be sure that plaques would be present at the time of the intervention, we used seven-month-old mice. On the other hand, at this age the plaques formed are not as dense as they can be in older mice, thus making it easier to identify the effect of continuous filtration treatment in a short-term study.

**Figure 1:**
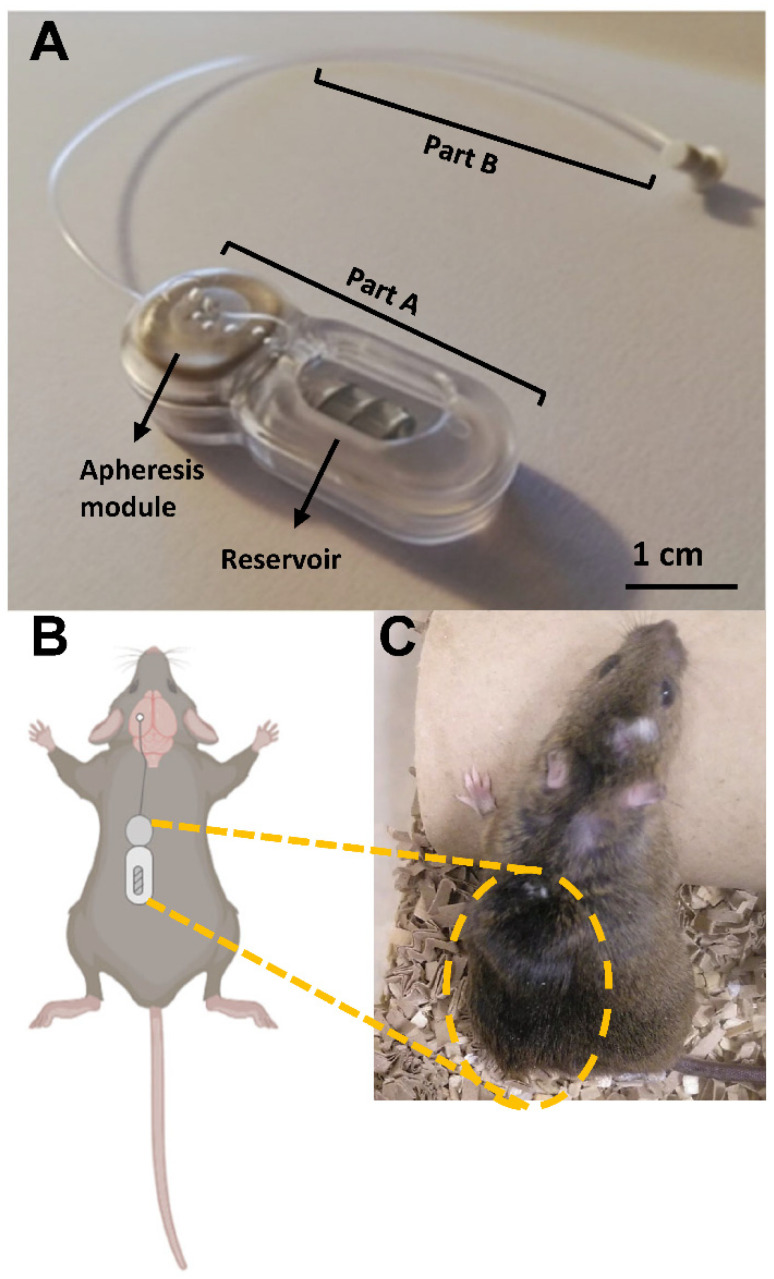
Components of the device for selective and continuous apheresis of CSF in mice. (**A**) Part A consists of the apheresis module where the nanoporous membrane for CSF filtration is located; coupled to this module is the reservoir where the therapeutic agent is contained. Both the apheresis module and the reservoir are protected by a capsule. Part B is composed of a cannula connected to the apheresis module and, at the other end, is coupled to a catheter that allows its implantation in the brain. (**B**) Scheme of the location of part A on the back of the mouse (made in BioRender.com), as well as its connection through part B to one of the cerebral hemispheres. (**C**) The whole system is implanted subcutaneously so that it remains protected from the normal activity of the mouse.

The miniaturized prototype of the implantable device consisted of an apheresis module with a reservoir (Part A; Figure 1A) and a catheter connecting Part A to a terminal cannula for intracerebroventricular implantation (Part B; Figure 1A). Part A of the device was placed subcutaneously on the back of the mouse, as was the cannula, and part B was implanted in the lateral ventricle (Figure 1B,C). Due to the weight (4.29 ± 0.41 g), but especially the size of the device (3.5 × 1 × 1 cm), only male mice were used in the study. In the case of the seven-month-old APP/PS1 mice, the weight of the device represents 12.86 ± 0.44% of the mouse’s weight, while in the case of Wt mice of the same age this percentage is 14.92 ± 0.38%. On the other hand, the most limiting factor was the length of the device, which, in relation to the length of the back of the mice, represents 66.51 ± 1.77% of the length in the case of the APP/PS1 mice and 69.56 ± 1.86% in the case of the Wt mice.

Considering this, a total of twenty-four male mice were successfully operated on, which seventeen were APP/PS1 and seven WT (Table 1). Reservoirs were filled with mAb in ten mice (two Wt and eight APP/PS1) and with vehicle (artificial CSF, aCSF) in ten mice (three Wt and seven APP/PS1). Among them, four mice were euthanized due to severe lethargy that appeared 48–72 h after surgery in three cases, while another mouse was sacrificed one week later. In addition, one mouse showed significant weight loss and was euthanized two weeks after surgery. The remaining sixteen mice showed no apparent complications and exhibited normal cage behavior, such as nest formation and grooming, reaching the end of the three-week infusion study. 

### 2.2. The Apheresis Module Is Permeable to Aβ and Impermeable to Anti-Aβ Antibody In Vivo

**Figure 2:**
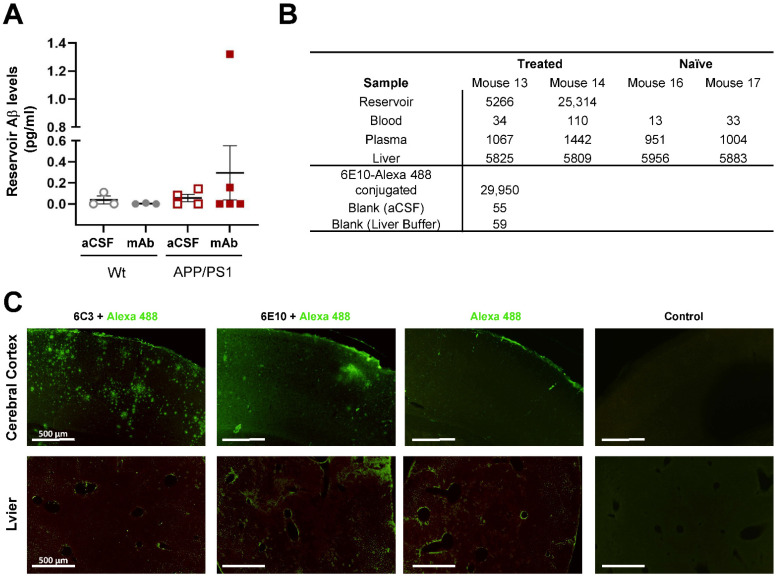
Permeability to Aβ and impermeability to mAb analysis after in vivo study. (**A**) Graph shows the levels of Aβ detected (pg/mL) in the reservoir of Wt and APP/PS1 mice, which could be loaded with vehicle (aCSF) or with anti-Aβ mAb. Data are presented as mean ± SEM. Each dot represents one mouse. (**B**) Emitted fluorescence data at 600 nm. Table shows the emission values at 600 nm detected in liquid samples from four APP/PS1 mice. These samples were obtained from the reservoir, whole blood, plasma, and liver homogenate from two mice with the device and treated with Alexa 488-conjugated mAb (clone 6E10) and two mice that had not been operated (naïve). Data for these mice can be found in Table 1. The 600 nm emission values for a 1:200 dilution of the mAb, as well as for aCSF used as vehicle and liver homogenization buffer are also shown. (**C**) Representative images of cerebral cortex and liver sections from APP/PS1 mice treated with Alexa 488-conjugated mAb (clone 6E10). From left to right, immunofluorescence in consecutive sections was developed using a primary antibody for Aβ plaque detection (clone 6C3), a primary antibody more specific for soluble Aβ (clone 6E10), only Alexa 488-conjugated secondary antibody, or no antibody at all. As shown in the images, no signal is detected in the absence of any antibody.

The nanoporous membranes (NPMBs) used in this study were first tested in vitro to determine their efficacy in terms of Aβ permeability (Supplementary Figure S1A,B). Their impermeability to molecules larger than the pore size of the NPMBs used here (9 ± 2 nm) was also tested with albumin (~68 kDa), which is smaller than mAb (~150 kDa), as a reference molecule (Supplementary Figure S1C,D). To determine whether the soluble Aβ peptide present in CSF was able to pass through the NPMBs in the apheresis module in vivo, Aβ levels within the reservoir were quantified with SIMOA after being explanted from sacrificed mice. One-third of the reservoirs analyzed showed the presence of Aβ in their content (Figure 2A; Table 1). 

On the other hand, the impermeability of the system to the therapeutic agent used in this study, anti-Aβ mAb (clone 6E10), was studied. For this purpose, four additional mice (two APP/PS1 and two WT) were treated with the Alexa 488-conjugated version of the same mAb (Table 1). The levels of fluorescence emitted by the mAb were then determined both in the reservoir and in different tissues of the mice. The table in Figure 2B shows fluorescence (emission: 600 nm) determined in the reservoir, in peripheral tissue (liver homogenate), and systemic blood (whole blood and plasma). As a control for the fluorescence emitted by the mAb, a 1:200 dilution was measured. The same dilution was also performed on the samples obtained from the reservoirs. In this way, it was determined that their fluorescence was higher than the emission levels at 600 nm detected in undiluted peripheral samples from the same mice. These samples also had similar emissions to the values detected in naïve mice, which were used as a control for basal autofluorescence. 

In addition, an immunofluorescence study was performed on cryopreserved sections of both the brain and liver (Figure 2C). First, we used a primary antibody that detects oligomeric forms of Aβ_42_ (clone 6C3) and a secondary antibody conjugated with Alexa 488. Results showed that, in the cerebral cortex, high abundance of Aβ plaques were detected, while no signal was found in the liver of these mice. In consecutive sections, another immunofluorescence was performed using the same primary antibody as for treatment (clone 6E10), with higher affinity for soluble and intracellular forms of Aβ [28]. In this case, slight labelling was observed in the cerebral cortex but not in the liver. However, incubation of the sections with only labelled secondary antibody or no antibody showed no signal in either the cerebral cortex or in the liver. All in all, these results indicate that the mAb contained in the reservoirs is not able to pass through the filtration system and therefore does not appear in brain tissue or peripheral tissues.

### 2.3. Continuous CSF Apheresis for Rapidly Decreased Area Covered by Aβ Plaques in the Cerebral Cortex

**Figure 3:**
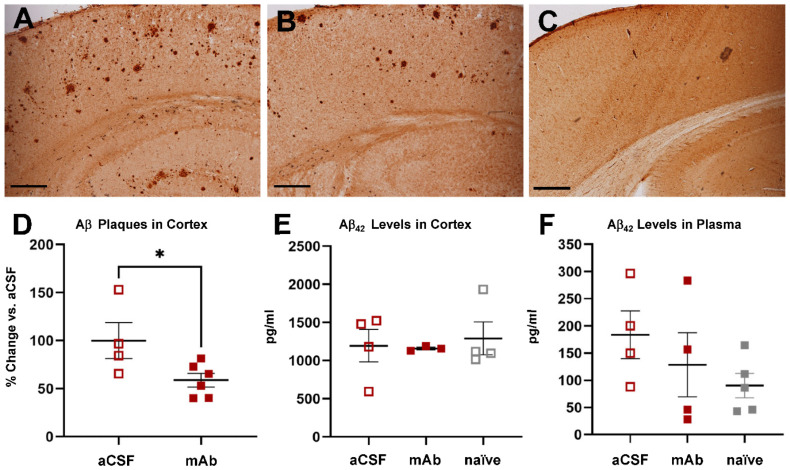
Evaluation of the effect of selective and continuous CSF apheresis in vivo. (**A**–**C**) Representative images of immunohistochemistry developed for the quantification of Aβ plaques in APP/PS1 mice with the device and treated with aCSF (**A**) or mAb (**B**) and their comparison with a Wt mouse (**C**). Scale bar: 100 µm. (**D**) Quantification of the area occupied by plaques with respect to the total surface area of the cortex is represented as the percentage of change with respect to aCSF-treated mice. Data are presented as mean ± SEM. Each dot represents one mouse. Statistical comparison between the two groups was performed using unpaired Student’s *t*-test. *, *p* < 0.05. (**E**) Graphical representation of the quantification of Aβ levels (pg/mL) in cerebral cortex homogenates from APP/PS1 mice with the device, containing aCSF or mAb, and in age-matched APP/PS1 mice that have not undergone surgery (naïve). Data are presented as mean ± SEM. Each dot represents one mouse. (**F**) Graphical representation of the quantification of Aβ levels (pg/mL) in plasma from APP/PS1 mice implanted with the device containing aCSF or mAb and in in age-matched APP/PS1 mice that have not undergone surgery (naïve). Data are presented as mean ± SEM. Each dot represents one mouse.

Once we determined that Aβ reaches the reservoir while the mAb remained retained in the filtration system in vivo, we assessed the effect of the three-week intervention. To this aim, we quantified the area occupied by Aβ plaques in relation to the total assessed area of the cerebral cortex in APP/PS1 mice (Figure 3). Results showed that continuous apheresis of CSF with anti-Aβ mAb significantly reduced the area occupied by plaques relative to aCSF APP/PS1 mice (100 ± 18.80% vs. 58.84 ± 7.01%; Figure 3A,B,D). In some of these mice, we also determined soluble Aβ_42_ levels in cerebral cortex homogenates and in plasma (Figure 3E,F). However, we observed no differences between mice treated with aCSF or mAb in relation to soluble Aβ_42_ levels in APP/PS1 mice of the same age and that had not undergone surgery at this level. Thus, these results indicated that continuous CSF apheresis reduces the surface area occupied by Aβ plaques in the cerebral cortex with no changes in brain or plasma soluble Aβ_42_ levels after this intervention time.

## 3. Discussion

In this work, we present a new implantable device for clearing selectively targeted molecules directly from the CSF. The innovative mechanism of action of this device is based on the property of selective molecular permeability of NPMBs. Thus, we bioengineered a modular device where the key component is the apheresis module endowed with tailored NPMBs with specific physicochemical properties. Additionally, we integrated the apheresis module with other components for access to the CSF and for infusion of therapeutic agents/aCSF. As the mechanism of action is based on the property of selective molecular permeability of NPMBs, we first tested the system both in vitro and in vivo, demonstrating the efflux of small molecules, such as Aβ, and the impermeability to molecules larger than the membrane pore size, such as albumin or immunoglobulins. Albeit we showed that membranes are permeable to Aβ, there were some inconsistencies among the different samples taken from the in vivo study that may relate to different variables, such as the use of therapeutic agent or aCSF, the respective doses, time of device implanted, and sample processing. Importantly, we have not assessed the level of biofouling that may have occurred in membranes and potentially affect the permeability properties over time [29]. Further studies are needed to better understand biofouling of membranes and the time-dependent molecular dynamics of Aβ and mAb through NPMBs in vivo. 

Even when implantation of the miniaturized prototype in mice is feasible and globally safe, complications may present in the immediate and deferred post-operatory. Some complications, such as weight loss and lethargy, are more likely to be derived from the surgical process itself or from the side effects of post-surgical medication than from the device or the therapeutic agent. However, the size of part A in the device is within the upper limit of what is feasible for an implantable subcutaneous device in mice. Therefore, we will work to fine-tune the device by mainly reducing the size of part A for future studies. In addition, we are currently conducting a thorough review of the protocols to increase safety and improve the welfare of the mice.

The main finding of this study is that continuous selective apheresis of soluble Aβ from the CSF rapidly clears amyloid plaques. This reduction could be due to the entrapment of Aβ into the apheresis module, thus generating an efflux from the ISF to the CSF. Previous studies have already reported that CNS-delivered immunotherapies with antibodies against Aβ, including the one used in this study, was effective in reducing both Aβ plaques and intracellular Aβ [28,30]. Those results were obtained either after an intrahippocampal injection in which case the reduction occurred on the ipsilateral side [30] or after an injection into the third ventricle with reduction observed bilaterally in the hippocampus but not in the amygdala [28]. Nevertheless, in both cases the effects were observed at seven days post-injection and were lost 30 days after injection. In this proof of concept, the reduction observed in up to three weeks of selective CSF apheresis confirms that this system elicits in the short time the same effect on Aβ plaques as Aβ-immunotherapies and other Aβ-clearing methods. However, theoretically, the more sustained the Aβ clearance effect, the more profound and extensive the reverse in the neuropathological and subsequent clinical changes will be in the long term. Consequently, systems for continuous and sustained clearance of target molecules in the CNS are needed, and the device here presented may contribute to this aim.

One of the main limitations of this study is that we have not performed functional studies to determine whether continuous and selective apheresis not only reduces Aβ plaques but also has cognitive benefits. However, a reduction of both plaques and intracellular Aβ has previously been reported to be associated with some cognitive impairments in murine models of AD [28]. Nevertheless, this is not the case in clinical trials with systemically administered anti-Aβ mAb which, while showing effective removal of plaques, no significant clinical benefit was found [13,14]. Indeed, plaque formation itself could be a defense mechanism [4], and densely deposited Aβ may not result as toxic as previously thought. Furthermore, the most toxic species of Aβ are oligomeric soluble forms [31,32], which is now perceived as an advantage of mAb specifically addressed against them [13]. Although in this study we have not detected changes in soluble Aβ in brain parenchyma or plasma, it is possible that longer apheresis times may have the capacity to modify the levels of these more toxic Aβ species. Therefore, in future long-term studies, a more comprehensive analysis of the different compartments into which soluble forms of Aβ may move will be needed to fully understand the dynamics of Aβ between plaques, ISF, CSF, and the apheresis module. 

Although the implantable device for CSF continuous and selective apheresis is an invasive procedure in small animal models, in its current form, the results obtained in this proof-of-concept study over a short period of time are promising. Cost/benefit implications of this therapeutic strategy for NND patients may be very high as this route provides several advantages over “standard” peripherally administered drugs, including: 1, the system allows acting on the CSF directly and continuously, providing a more powerful clearance effect than therapies based on peripheral clearance; 2, being a selective therapy prevents potential safety issues that may occur if the levels of other molecules in the CSF were modified; 3, *immunoisolation* of mAb impedes immune responses, fully avoiding one of the most serious side effects reported with mAb systemically administered. All in all, the innovation here presented opens up the door to a substantial improvement in the treatment of devastating NDD for which there is currently no cure.

## 4. Materials and Methods

### 4.1. Mice

Seventeen seven-month-old male APP/PS1 mice, transgenic for mouse/human amyloid precursor protein (Mo/HuAPP695swe) and a mutant human presenilin 1 (PS1-dE9) in 129Sv background and seven non-transgenic littermates (Wt) were used for device implantation. These mice were randomly divided into two groups: control (vehicle, aCSF) and treatment (mAb). Nine additional male mice of the same age, but which had not undergone surgery, were also used (eight APP/PS1 and one Wt; naïve). The distribution of these mice in the different groups and their use for the respective procedures can be seen in Table 1.

Mice were maintained with food and water *ad libitum* on a 12 h light/dark cycle (onset at 8:00 a.m.) and under controlled temperature (22 ± 2 °C). All procedures were conducted during the light portion of the cycle between 8:30 and 14:00 a.m. at the Animal Facility of Universidad de Oviedo and were performed in accordance with institutional guidelines approved by The Research Ethics Committee of the University of Oviedo (PROAE 32/2020).

### 4.2. Implantable Device for Mice

We have designed a prototype of an implantable device for continuous and selective apheresis of CSF (Figure 1A). The device was miniaturized to adapt it to the size of mice and to manufacture in biocompatible materials. It consists of two main components, a subcutaneous reservoir and an apheresis module endowed with tailored NPMBs [33,34], which were encapsulated together in a capsule made by Neuroscience Innovative Technologies (Figure 1A, part A). For this study, NPMBs have a pore size of 9 ± 2 nm. This allows the passive flow of smaller substances, such as soluble Aβ (~4 kDa in its monomeric form), on both sides while preventing the passage of molecules larger than 10 nm, e.g., antibodies (immunoglobulin G, ~150 kDa) or albumin (~68 kDa) that may act as therapeutic agents (Supplementary Material). These agents are retained in the device within the subcutaneous reservoir which has a volume of 100 µL. At the top of the reservoir is a self-sealing port, which allows filling using a 50 µL syringe (800 series microliter syringes; Hamilton, Reno, NV, USA). This design allows drugs confined in the reservoir to interact with the target molecule that is filtered through the NPMBs.

The apheresis module is connected through a brain infusion cannula (Brain Infusion kit 2; Alzet, Cupertino, CA, USA) to one of the lateral ventricles of the brain (Figure 1A, part B).

### 4.3. Filling the Reservoir with Anti-Aβ mAb or Vehicle

The reservoir was filled under sterile conditions with an anti-Aβ_1–42_ mAb, which recognizes human but not murine Aβ_1–42_. To estimate the most appropriate dose of antibody, we considered previous studies in which a single dose of antibody was injected intracerebroventricularly, and Aβ clearance was evaluated [28]. Since these studies showed that the dose applied, 10 µg, had an effect up to seven days but not up to thirty days, we considered a dose of 50 µg of antibody and a study time of three weeks in our experiment to assess the changes associated with continuous CSF apheresis in the presence of this therapeutic agent. Thus, ten devices (Table 1) were loaded with a solution of non-conjugated anti-β-Amyloid antibody (clone 6E10; Biolegend, San Diego, CA, USA) diluted in artificial CSF (aCSF; ACSF, Tocris Bioscience, Bristol, UK). Additionally, another four were filled with fluorophore-conjugated anti-Aβ antibody (Alexa Fluor^®^ 488 anti-β-Amyloid, clone 6E10; Biolegend). Final mAb concentration within the device was 500 µg/mL. As control, ten devices were filled with the same volume of aCSF. 

### 4.4. Surgery and Mice Welfare

The surgical procedure was developed according to the protocol described in [35]. Briefly, anesthetized mice (2% isofluorane and 0.4 L/min O_2_) were placed in the stereotaxic frame (Digital Compact Mouse Stereotaxic Instrument; Harvard Apparatus, Holliston, MA, USA), and the surgery area was then shaved. To expose the skull, an incision was made from the middle of the skull to the base of the neck from where a subcutaneous pocket was opened, and the device was inserted. Finally, the cannula was implanted into the left lateral ventricle. Surgery coordinates, with respect to Bregma, were AP—1.26 mm, ML—0.7 mm, and—2.5 mm to the skull. The cannula was glued with cyanoacrylate (Cicastik Suturvet; Chemical Iberica, Salamanca, Spain), and the incision was sutured using 5–0 suture thread (Sterile Surgical Suture; LorcaMarín, Murcia, Spain). 

After surgery, all the mice were placed in an intensive care cage with exhaustive temperature control (28–30 °C; Vetario S50, Kanalvet, Bizkaia, Spain) during the first 24 h to ensure proper recovery. All mice received pre- and post-surgical analgesic and antibiotic medication; additionally, they were housed individually after surgery to prevent damage to the sutures.

Termination was scheduled at 21 days. Throughout the study, the mice were monitored daily, observing their weight, cage behavior (nest formation), food, and water consumption. 

### 4.5. Tissue and Fluids Collection and Processing

Three weeks after surgery—or earlier if severe complications presented—mice were deeply anaesthetized with ketamine (100 mg/kg) and xylazine (10 mg/kg) in saline solution. The device was carefully removed and thoroughly cleaned externally with 70% ethanol. Subsequently, the fluid was removed from the reservoir and stored at −80 °C for further study. Peripheral blood samples were collected from the inferior vena cava (~0.5–1 mL). For this, EDTA (as anticoagulant; 1.5 mg/mL blood) pretreated 1 mL syringes (BD, Franklin Lakes, NJ, USA) and intravenous catheters (BD Insyte-W, 21 GA, BD) were used. Blood was immediately centrifuged at 2000 g, and plasma was stored at −80 °C until use. After blood extraction, the abdominal aorta was cut at the same level of the vena cava, and sterile ice-cold phosphate buffer saline (PBS; ~30 mL) was perfused to clean tissues.

#### 4.5.1. Tissue Processing for Biochemical Analysis

In mice used for biochemical analysis, the brains were sagittally dissected, and the cerebral cortexes were extracted from the right hemispheres and subsequently frozen at −80 °C. In addition, liver portions (equal to 200 mg) were removed and split into two halves, one of which was also frozen until use at −80 °C. 

The cerebral cortexes and livers (100 mg) were homogenized in lysis buffer (20 mM HEPES pH 7.4, NaCl 100 mM, NaF 50 mM, EDTA 5 mM, Triton X-100 1%) with protease inhibitors (cOmplete™, Mini Protease Inhibitor Cocktail; Roche, Basel, Switzerland) in a glass potter homogenizer on ice. Subsequently, samples were centrifuged for 10 min at 12,000× *g* at 4 °C, and the supernatants were collected for analysis. Total protein concentration was determined using NanoDrop One 2000c, Spectrophotometers (Thermo Scientific, Waltham, MA, USA). 

#### 4.5.2. Tissue Processing for Histological Analysis

The left hemisphere and liver tissues were fixed with 4% paraformaldehyde (PFA; pH 7.4) and freshly prepared from 16% PFA (Electron Microscopy Science, Hatfield, PA, USA) diluted in Sorensen’s phosphate buffer overnight at 4 °C for histology processing. Then, they were washed in PBS and immersed in 30% sucrose in PBS for 24 h for cryoprotection. Samples were then frozen in OCT (Optimal Cutting Temperature, Tissue-Tek) and stored at −80 °C until use. Coronal sections (40 μm) were cut on a cryostat (Leica CM1900, Leica Microsystems, Wetzlar, Germany) and preserved in a solution containing 30% glycerol and 30% ethylene glycol in 0.02 M monobasic phosphate buffer of pH 7.2 at −20 °C until further use.

### 4.6. Quantification of Cerebral Cortex Aβ Plaques

For free-floating immunohistochemistry, ten non-consecutive coronal sections (spaced 300 µm) for each mouse were used. After washing with 0.1 M monobasic phosphate buffer (PB; pH 7.4), a heat-mediated citrate buffer pH 6.0 antigen reveal step was performed. Next, the slices were washed with PB, and the peroxidase endogenous activity was quenched for 45 min with 1% H_2_O_2_. Sections were then washed with PBS and incubated with 1% Triton X-100, 1% bovine serum albumin (BSA), and 0.5% fetal bovine serum (FBS). For Aβ staining, samples were incubated with anti-Aβ antibody [MOAB-2] (1:200, clone 6C3, Abcam) under gentle shaking at 4 °C for four days. Peroxidase activity was developed with the Vectastain Elite ABC-HRP kit (Rabbit IgG; Vector Laboratories) using diaminobenzidine (DAB; Abcam, Cambridge, UK).

Images corresponding to the cerebral cortex were obtained with an Olympus BX61 microscope (4× objective) equipped with a digital camera (Olympus D70). Photomicrographs were analyzed using ImageJ software [36]. To obtain the percentage of area occupied by Aβ plaques in cortex, we applied the following formula for each slice: [area covered with Aβ plaques in cortex]/[area of cortex]. The mean of each mouse was then calculated, and subsequently, the average of each of the treatment groups was determined. 

### 4.7. Fluorescence Analysis

#### 4.7.1. Immunofluorescence

For free-floating immunofluorescence, consecutive coronal sections of hemibrains and liver sections for each mouse were used. After washing with PB, sections were then washed with PBS and incubated with 1% Triton X-100 and 1% BSA. For Aβ staining, samples were incubated with anti-Aβ amyloid antibody [MOAB-2] (1:200, clone 6C3, Abcam) or anti-β-amyloid (1:1000; clone 6E10, Biolegend) under gentle shaking at 4 °C for four days. After this, slices were incubated with the secondary antibody conjugated with Alexa 488 (Donkey anti-Mouse IgG (H + L) Alexa Fluor Plus 488; Invitrogen, Hong Kong, China). Sections incubated only with the secondary antibody or without primary or secondary antibodies were used as controls. Sections were incubated with DAPI and then mounted on slides with polyvinyl alcohol mounting medium with DABCO (Supelco, Bellefonte, PA, USA). Then, they were observed with an Olympus BX61 fluorescence microscope. 

#### 4.7.2. Fluorescence Levels

Levels of fluorescence were measured in whole blood, plasma, and liver homogenates. A total of 50 µL of samples were aliquoted into a black 96-well optical bottom plate (Fisher Scientific, Hampton, NH, USA). The plate was scanned on an Odyssey XF Imaging System (LI-COR Biosciences, Lincoln, NE, USA) and expressed as mean fluorescence (signal).

### 4.8. Aβ_1–42_ Immunoassay 

Aβ_1–42_ levels in plasma and reservoir fluid were measured on the ultra-sensitive single-molecule array (Simoa) HD-X analyzer platform (Quanterix, Billerica, MA, USA) following the manufacturer’s instructions. The Aβ_1–42_ Simoa 2.0 assay (Cat. N° 101664) was purchased from Quanterix. This assay measures levels of Aβ_1–42_ in human plasma with a detection limit of 0.044 pg/mL (range 0.0017–0.108 pg/mL). Two quality control samples were run at the same time as the samples. Calibrators and plasma samples were run in duplicates, and the average of the two measurements (pg/mL) was used for statistical analysis. Further, Aβ_1–42_ levels in brain cortex homogenates were quantified by ELISA kit following manufacture instructions (Human Aβ_42 ELISA Kit; Invitrogen, Waltham, MA, USA).

### 4.9. Statistical Analysis

If not otherwise indicated, data are presented as mean ± SEM. GraphPad Prism 9.4.0 (GraphPad Software, Dotmatics, Bishop’s Stortford, U.K.) was used for statistical analysis and graphical representation. Normality of the variables was determined using the Shapiro–Wilk test. In the case of the measurement of Ab levels in the reservoir and in cerebral cortex homogenates, comparison among groups was performed using the non-parametric Kruskall–Wallis test. For plasma Aβ levels, this comparison was conducted using ANOVA. Finally, quantification of Aβ plaques in cerebral cortex was done using Student’s *t*-test for independent samples to compare the aCSF and mAb groups. A critical value for the significance of *p* < 0.05 was considered throughout the study.

## 5. Conclusions

We present a new system for clearing selectively target molecules directly from the CSF based on the property of selective molecular permeability of NPMBs that might be suitable for treating NDD where pathogenic molecules are identified, soluble, and present in the CSF. 

We provide the first proof of concept in a mice model of AD where we found that continuous clearance of soluble Aβ rapidly decreases cortical amyloid plaques.

This therapeutic strategy is feasible and may provide important advantages in terms of safety and efficacy. However, fine-tuning the device, refining the implantation procedures, and more studies over longer periods of time are needed to further observe safety, efficacy, and the potential biofouling of the system before progressing this innovation to more advanced technical readiness levels.

## Figures and Tables

**Table 1:** Summary of the different procedures or techniques carried out on each of the mice used.

Mice ID	Genotype	Content Infused	Time Implanted	Complications	Aβ Permeability				mAb Impermeability	
					Aβ Reservoir	Aβ Plasma	Aβ Cortex	IHC	IF	Fluorescence
7	APP/PS1	mAb	48 h	Lethargy	+	+				
20	Wt	mAb	48 h	Lethargy						
21	Wt	aCSF	72 h	Lethargy	+					
5	APP/PS1	mAb	1 week	Lethargy				+		
11	APP/PS1	mAb	2 weeks	Weight loss	+		+	+		
26	APP/PS1	mAb	2 weeks	Lethargy			+			
27	APP/PS1	aCSF	2 weeks	Lethargy			+			
28	APP/PS1	aCSF	2 weeks	Lethargy			+			
2	APP/PS1	aCSF	3 weeks	None	+	+		+		
8	APP/PS1	aCSF	3 weeks	None	+	+	+	+		
9	APP/PS1	aCSF	3 weeks	None	+	+		+		
12	APP/PS1	aCSF	3 weeks	None	+	+	+	+		
4	APP/PS1	aCSF	3 weeks	None	+	+				
17	Wt	aCSF	3 weeks	None	+	+				
19	Wt	aCSF	3 weeks	None	+	+				
3	APP/PS1	mAb	3 weeks	None	+	+		+		
6	APP/PS1	mAb	3 weeks	None	+	+		+		
10	APP/PS1	mAb	3 weeks	None	+	+	+	+		
1	APP/PS1	mAb	3 weeks	None		+		+		
18	Wt	mAb	3 weeks	None	+					
13	APP/PS1	mAb 488	3 weeks	None					+	+
14	APP/PS1	mAb 488	3 weeks	None					+	+
15	Wt	mAb 488	3 weeks	None	+	+				
16	Wt	mAb 488	3 weeks	None	+	+	+			
29	APP/PS1	naïve	-	None		+				
30	APP/PS1	naïve	-	None					+	+
31	APP/PS1	naïve	-	None					+	+
32	APP/PS1	naïve	-	None		+	+			
33	APP/PS1	naïve	-	None		+	+			
34	APP/PS1	naïve	-	None		+				
35	APP/PS1	naïve	-	None		+	+			
36	Wt	naïve	-	None		+				
37	APP/PS1	naïve	-	None			+			

## Data Availability

The data that support the findings of this study are available from the corresponding author, MMG, upon reasonable request.

## Supplementary Materials

The following supporting information [37] can be downloaded at: https://www.mdpi.com/article/10.3390/ijms23169256/s1.
