# Supplementary material for: New, Fully Implantable Device for Selective Clearance of CSF-Target Molecules: Proof of Concept in a Murine Model of Alzheimer’s Disease
Source: Int J Mol Sci. 2022 Aug 17;23(16):9256. doi: 10.3390/ijms23169256 (PMC9408974; doi:10.3390/ijms23169256)
Supplement: Supplementary file 1 [file ijms-23-09256-s001.zip › ijms-1866485-supplementary.pdf]

Supplementary Materials for

New fully implantable device for selective clearance of CSF-target molecules: proof-of-concept in a murine model of Alzheimer's disease

María Almudena Coto-Vilcapoma[1,2 *], Juan Castilla-Silgado[1,2, *], Benjamín Fernández-García[2,3], Paola Pinto-Hernández [1], Raffaela Cipriani[4], Estibaliz Capetillo-Zarate[4,5], Manuel Menéndez-González[2,6,8,#], Marco Álvarez-Vega[7,9,§], Cristina Tomás-Zapico[1,2,§]

Correspondence to: menendezgmanuel@uniovi.es

This document includes:

Supplementary materials and methods

Supplementary results

Figure S1

Supplementary references

Supplementary materials and methods

Fabrication of nanoporous membranes

Nanoporous membranes (NPMBs) were obtained by electrochemical anodization from aluminum foils according to the two-step anodization method followed by atomic layer deposition (ALD) with SiO_2 coating as previously described [1]. SME and transmission spectra showed structures with a homogeneous pore size (9 ± 2) nm and porosity (7 ± 2)% [1].

Assessing the selective molecular permeability in vitro

To perform selective permeability studies of NPMBs, homemade permeation chambers were prepared. For this purpose, 6-well plates with culture plate inserts (VWR) were used and the membranes of the inserts were replaced by our NPMBs. With this design, wells act as donor cells, whereas inserts serve as receptor cells.

To determine the permeability of Aβ through NPMBs, human recombinant $A\beta_{1-42}$ (Abcam) was used. Films of $A\beta_{1-42}$ in HFIP (1,1,1,3,3,3-Hexafluoro-2-propanol, Merck) resuspended in a buffer containing 40 mM HEPES and 200 mM NaCl were prepared as previously described [2]. $A\beta_{1-42}$ concentration on films was determined by BCA (Pierce BCA Protein Assay kit; Thermo Scientific). Donor or receptor cells were filled with aCSF (Tocris Bioscience) or with a supraphysiological dose of $A\beta_{1-42}$ (20 µg/ml). The opposite cells of the same permeation chamber were filled with aCSF. Controls permeation chambers were also used, where only $A\beta_{1-42}$ solution was added and no NPMB was present. Permeation chambers were then incubated in a humidified chamber with 5% CO_2, at 37 °C for 72 hours. $A\beta_{1-42}$ concentration at this endpoint was determined in both donor and receptor cells by ELISA following manufacturer's instructions (Human Aβ_42 ELISA Kit; Invitrogen).

To analyze selective permeability of NPMBs, bovine serum albumin fraction V (~ 68 kDa; PanReac AppliChem) was used. Similar to $A\beta_{1-42}$ study, donor or receptor cells were filled with aCSF or with a supraphysiological CSF dose of albumin (2 mg/ml). The

opposite cells of the same permeation chamber were filled with aCSF. Controls permeation chambers were also used, where only albumin solution was added and no NPMB was present. Permeation chambers were then incubated for 72 hours. Albumin concentration at this endpoint was determined in both donor and receptor cells by BCA.

Supplementary results

To determine whether fabricated NPMBs allowed permeation of substances smaller than their pore size (~9 nm) to pass though them in both directions, permeation chambers separating two cells, donor and receptor, were prepared. We used recombinant human $A\beta_{1-42}$ peptide, which in its monomeric state has a size of 4 kDa, as the model substance (Figure S1A). To prevent $A\beta_{1-42}$ aggregation during the study, we used a previously described protocol for the resuspension of $A\beta_{1-42}$ that prevents high oligomerization. After adding $A\beta_{1-42}$ to either the donor cell or the recipient cell, the levels of $A\beta_{1-42}$ in both cells were determined after 72 hours of incubation in the permeation chambers. The results showed that both cells had the same amount of $A\beta_{1-42}$, regardless of which cell had $A\beta_{1-42}$ at the beginning of the study (Figure S1B). This indicates that the designed NPMBs had a pore size suitable for the efflux of small substance, such as soluble $A\beta_{1-42}$.

Once this was determined, we assessed whether this permeability is indeed selective, using a molecule much larger than the pore size of the NPMBs, such as albumin (~ 68 kDa; Figure S1C). Thus, albumin was added to either the recipient cell or the donor cell and its concentration in both cells was analyzed after 72 hours of incubation. Results revealed that albumin was detected in those cells where it was first placed, indicating that the membranes do not allow substances larger than the nanoporous size to pass through. Thus, the designed NPMBs are permeable to small molecules, such as $A\beta_{1-42}$, and impermeable to larger molecules, such as albumin.

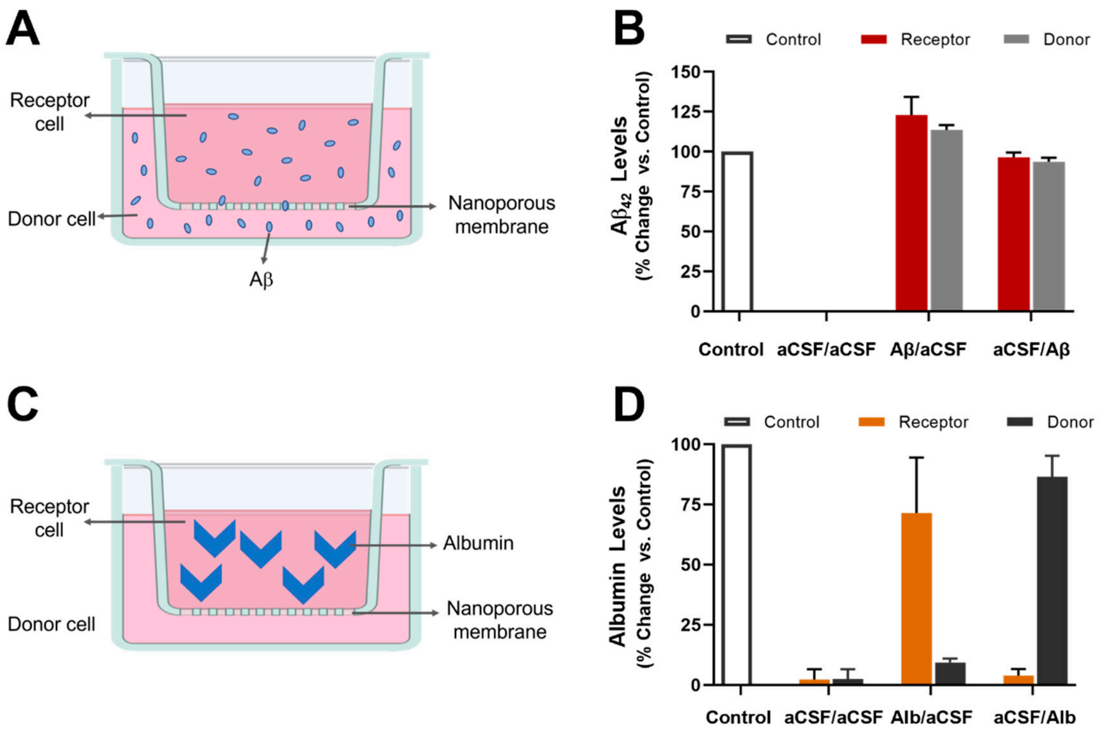

Figure S1. ***In vitro* testing of nanoporous membranes efficacy**. **A.** Scheme of a permeation chamber, where the donor cell is separated from the receptor cell by a nanoporous membrane. By adding Aβ into the donor or the receptor cell, it is expected that the peptide diffuses through the nanoporous membrane. **B.** 72-hour time course study of Aβ permeability on both sides of the nanoporous membrane. Graph shows Aβ levels expressed as a percentage regarding control permeation chamber, where no nanoporous membrane was present. Receptor cell/Donor cell. aCSF (artificial CSF). Data are represented as mean ± SEM (n=2 replicates). **C.** Scheme of a permeation chamber, where a molecule, such albumin, larger than the porous size of the membrane is added. If albumin is added in either the receptor or the donor cell, it cannot flow from one to the other. **D.** 72-hour time course study of albumin impermeability on both sides of the nanoporous membrane. Graph shows albumin levels expressed as a percentage regarding control permeation chamber, where no nanoporous membrane was present. Receptor cell/Donor cell. Alb (albumin). Data are represented as mean ± SEM (n=6 replicates).

Supplementary references

1. Gonzalez, A.S.; Vega, V.; Cuevas, A.L.; Yuso, M.; Prida, V.M.; Benavente, J. Surface Modification of Nanoporous Anodic Alumina during Self-Catalytic Atomic Layer Deposition of Silicon Dioxide from (3-Aminopropyl)Triethoxysilane. *Materials (Basel)* **2021**, *14*, doi:10.3390/ma14175052.
2. Krishtal, J.; Metsla, K.; Bragina, O.; Tougu, V.; Palumaa, P. Toxicity of Amyloid-beta Peptides Varies Depending on Differentiation Route of SH-SY5Y Cells. *J Alzheimers Dis* **2019**, *71*, 879-887, doi:10.3233/JAD-190705.
